# Influence of indigenous non-*Saccharomyces* yeast strains on the physicochemical and sensory properties of wine fermentation: a promising approach to enhancing wine quality

**DOI:** 10.3389/fcimb.2024.1495177

**Published:** 2024-12-06

**Authors:** Sathivel Thivijan, Dayani Pavalakumar, Chathuri J. Gunathunga, Lanka J. S. Undugoda, Pathmalal M. Manage, Ruwani N. Nugara, Pasan C. Bandara, Kasun M. Thambugala, Fahad Al-Asmari, Itthayakorn Promputtha

**Affiliations:** ^1^ Department of Biology, Faculty of Science, Chiang Mai University, Chiang Mai, Thailand; ^2^ Department of Biosystems Technology, Faculty of Technology, University of Sri Jayewardenepura, Homagama, Sri Lanka; ^3^ Faculty of Graduate Studies, University of Sri Jayewardenepura, Nugegoda, Sri Lanka; ^4^ Centre for Water Quality and Algae Research, Department of Zoology, Faculty of Applied Sciences, University of Sri Jayewardenepura, Nugegoda, Sri Lanka; ^5^ Genetics and Molecular Biology Unit, Faculty of Applied Sciences, University of Sri Jayewardenepura, Nugegoda, Sri Lanka; ^6^ Center for Biotechnology, Department of Zoology, University of Sri Jayewardenepura, Nugegoda, Sri Lanka; ^7^ Center for Plant Materials and Herbal Products Research, University of Sri Jayewardenepura, Nugegoda, Sri Lanka; ^8^ Department of Food and Nutrition Sciences, College of Agricultural and Food Sciences, King Faisal University, Al Ahsa, Saudi Arabia

**Keywords:** fermentation kinetics, *Hanseniaspora uvarum*, HPLC, sensory attributes, *Vitis vinifera*

## Abstract

This study explores the potential of indigenous non-*Saccharomyces* yeasts isolated from *Vitis vinifera* L. grape skins to improve the quality of regional wines by enhancing their physicochemical and sensory characteristics. Five promising yeast strains were identified at different stages of fermentation: *Hanseniaspora opuntiae* (J1Y-T1), *H. guilliermondii* (Y5P-T5), *H. uvarum* (JF3-T1N), *Pichia kudriavzevii* (Y8P-T8), and *Starmerella bacillaris* (WMP4-T4). Among these, *H. uvarum* and *S. bacillaris* were particularly noteworthy due to their superior alcohol production, achieving levels of 8.16 ± 0.05% and 8.04 ± 0.04% (v/v), respectively, and demonstrating higher alcohol tolerance even in later fermentation stages. *Hanseniaspora uvarum* also showed exceptional resilience, with a half-life of 3.34 ± 0.03 days and a Km value of 1.0200 ± 0.0100 mol L⁻¹, achieving the highest biomass even in the later stages of fermentation. High-Performance Liquid Chromatography analysis revealed that while tartaric acid levels remained constant, malic acid content decreased, and acetic acid was produced by all strains. Solid-Phase Microextraction-Gas Chromatography Mass Spectrometry identified ethyl acetate as the dominant volatile compound, with *H. uvarum* producing the highest concentration (43.411 ± 1.602%), contributing to a fruitier aroma and flavor. The combined attributes of *H. uvarum* higher alcohol content, enhanced fruity notes, improved clarity, lower acetic acid (0.52 ± 0.03 g L⁻¹), and significant residual sugar (162.37 ± 2.48 g L⁻¹) make it a promising candidate for improving the overall quality of regional wines. Incorporating *H. uvarum* into mixed starter cultures with specific *Saccharomyces* strains could further optimize the wine fermentation process.

## 1 Introduction

The quality of wine is significantly influenced by the Wine Microbial Consortium (WMC), which comprises various microorganisms, including yeasts, lactic acid bacteria, and acetic acid bacteria (Camilo et al., 2022). The wine production process begins with the harvest of grapes and progresses through multiple fermentation stages, where the interactions among these microbial populations play a crucial role in shaping the final product’s sensory and chemical attributes (Van Leeuwen and Seguin, 2006; Bokulich et al., 2014; Nardi, 2020). The composition of the WMC is affected by several factors, including grape cultivar, ripeness, climate conditions, and vineyard management practices (Čadež et al., 2010; Pinto et al., 2014; Yao, 2023).

Although *Saccharomyces cerevisiae* is the dominant yeast in wine fermentation, its isolation from mature, healthy grapes can be challenging (Capece et al., 2013). Initially, a diverse array of yeast communities including *Candida*, *Hanseniaspora*, *Issatchenkia*, *Kluyveromyces*, *Metschnikowia*, and *Pichia* are present, but *S. cerevisiae* takes over in the later stages of fermentation (Ribereau-Gayon et al., 2005; Ciani and Comitini, 2019; Fazio et al., 2023). The dynamics of these fungal microbiomes are critical, with specific genera like *Hanseniaspora uvarum* being prevalent during the fermentation process (Onetto et al., 2024). These communities exhibit significant temporal and spatial variability, affecting fermentation outcomes. Microbial populations metabolize grape sugars, producing secondary metabolites and enzymes that significantly impact the sensory properties of the wine (Fleet, 2003; Tofalo et al., 2021; Romano et al., 2022). Notably, non-*Saccharomyces* yeasts enhance the hydrolysis of monoterpene glycosides, thereby influencing aroma profiles (Schober et al., 2023).

The wine industry is increasingly investigating non-*Saccharomyces* yeasts for their ability to impart distinct flavors and aromas by converting organic compounds into specific volatile compounds during fermentation (Tofalo et al., 2011; Steensels and Verstrepen, 2014; Berbegal et al., 2020; Tufariello et al., 2021). The introduction of new strains from grapes and fermented musts can deepen our understanding of fermentation dynamics and support the development of innovative starter cultures (Fleet, 2008; Ciani et al., 2010; Zhu et al., 2021). Specific enzymatic activities exhibited by these yeasts, such as glycosidases, are essential for releasing volatile terpenes and other aroma compounds, further enhancing wine complexity (Romano et al., 2022).

Traditionally isolated from grape berries and juice, non-*Saccharomyces* yeasts have also been found in spontaneously fermented musts, showcasing their vitality during fermentation processes (Combina et al., 2005; Cordero-Bueso et al., 2013; Vilela et al., 2020). Studies have highlighted strains such as *Torulaspora delbrueckii* and various *Hanseniaspora* species for their positive contributions to fermentation dynamics and aromatic complexity (Borren and Tian, 2020). Their presence has been associated with improved sensory evaluations and the release of varietal aromas (López-Enríquez et al., 2023; Duka et al., 2024). Co-culturing non-*Saccharomyce*s yeasts with *S. cerevisiae* has demonstrated that they can withstand higher levels of alcohol, sulfur dioxide, and sugar concentrations, enhancing their viability in challenging fermentation environments (Maicas and Mateo, 2023). As fermentation progresses, *S. cerevisiae* produces ethanol, limiting the growth of other microbes, while tartaric acid acts as a growth inhibitor for *S. cerevisiae*, creating a stable fermentation context (Fleet, 2008; Albergaria and Arneborg, 2016).

Effective isolation of non-*Saccharomyces* yeast strains occurs during the early stages of fermentation, with species such as *Candida*, *Hanseniaspora*, *Metschnikowia*, *Lachancea* (formerly *Kluyveromyces*), *Pichia*, and *Saccharomyces* participating in spontaneous wine fermentation driven by the native WMC (Cocolin et al., 2000; Mills et al., 2002; Fleet, 2008; Maicas and Mateo, 2023). Major wine-producing countries utilize commercial strains of *S. cerevisiae* alongside non-*Saccharomyces* yeasts to create distinctive wines (Franco et al., 2021). Recent studies have revealed regional distributions of WMC in various countries, contributing to our understanding of wine microbiome dynamics and biodiversity (Setati et al., 2012; Bokulich et al., 2014; Taylor et al., 2014; De Gioia et al., 2022).

Despite extensive research on *Saccharomyces* strains in wine fermentation, the exploration of non-*Saccharomyces* yeasts, particularly their dynamics and biodiversity in Sri Lanka, remains largely unexplored. Although industrial-scale wine production is limited in Sri Lanka, there is a growing interest among small-scale winemakers to showcase unique Sri Lankan wine flavors on the global stage. Therefore, this study aims to isolate efficient wild-type non-*Saccharomyces* yeasts from specific geographical regions of Sri Lanka using Israel blue grapes (*Vitis vinifera* L.) and to correlate their microbial profiles with the sensory properties of the produced wine.

## 2 Materials and methods

### 2.1 Sampling, isolation, and identification of non-*Saccharomyces* yeasts

Initially, healthy, undamaged Israel blue grape (*Vitis vinifera* L.) samples were collected from various districts in Sri Lanka: Jaffna District (Urumpirai), Kilinochchi District (Palai), Puttalam District (Kalpitiya), Anuradhapura District (Mahailuppallama) and Kandy District (Kundasale).

Non-*Saccharomyces* yeasts were isolated from the collected grapes using two sample types; fresh grape skin, and different fermentation stages (early, middle, and late) of grape must. The grape skin was washed with saline solution (0.9%) in a shaker at 120 rpm for 15–20 minutes to extract the microorganisms from the grape skin. For the fermentation technique, grapes were crushed and allowed to undergo spontaneous fermentation. Microorganisms were then isolated from the early (1st day), middle (7th day), and final (14th day) stages of fermentation using YPD (Yeast extract peptone dextrose, HiMedia, India), and TSA (Tryptic soya agar, HiMedia, India) mediums and incubated at 28-30°C for 48 hrs (Kántor et al., 2017). Morphological characterization and budding formations were observed under microscopic view. To check the fermentation ability and CO_2_ production of the isolates, phenol red glucose broth with Durham tubes was used (Barnett et al., 2000).

### 2.2 Molecular identification

DNA extraction of selected yeasts was carried out using a Wizard genomic DNA purification kit (Promega, USA). The polymerase chain reaction (PCR) was then performed using a thermocycler (BIOER, LifeECO) with universal primers for the internal transcribed spacer (ITS) region: ITS1 (5´-TCC GTA GGT GAA CCT TGC GG-3´), and ITS4 (5´-TCC TCC GCT TAT TGA TAT GC-3´) to amplify the ITS region of the yeast rDNA (Kurtzman and Robnett, 1997). The PCR parameters were as follows: for the ITS amplification, the procedure begins with an Initial Denaturation step, where the reaction is heated to 95 °C for 5 minutes. This is followed by Cycling Steps repeated 35 times, consisting of Denaturation at 95 °C for 1 minute, Annealing at 52 °C for 45 seconds, and Extension at 72 °C for 1 minute. After the cycles, a Final Extension at 72 °C for 7 minutes is performed. The procedure concludes with a Final Hold at 40 °C to stabilize the PCR products for further analysis (Barata et al., 2012). Finally, PCR products were sent to Macrogen Inc., Korea for sequencing. Furthermore, phylogenetic characterization was carried out by comparing the sequences with previously identified non-*Saccharomyces* yeasts from other countries through BLASTN search of GenBank.

### 2.3 Substrate utilization and fermentation kinetics of isolated yeast strains

The thermovinification process was carried out on the fresh grapes. This technique involves heating grapes to temperatures between 60–80°C for 20–30 minutes, which facilitates the release of anthocyanins and other phenolic compounds from the grape tissues (Hanamant et al., 2015). After cooling the grapes naturally, they were then crushed aseptically. Subsequently, 1 mL of each identified non-*Saccharomyces* yeast strain (10⁸ CFU mL^-1^) was inoculated separately into 200 mL of grape must and allowed to ferment at room temperature. Fermentation for each strain was performed in triplicate under the same conditions. No additives were added to the must during fermentation.

#### 2.3.1 Residual sugar analysis

To analyze the substrate, residual sugar in the must and wine was calculated. Using the total soluble solids measured with a Brix meter (RHB-32ATC, ERMA, Tokyo) and the density of the wine during fermentation (monitored by weight and volume), residual sugars were determined following the model developed by Yaa’ri et al. (2024).

#### 2.3.2 Alcohol level determination

To determine the produced alcohol level, standard solutions containing 10% ethanol and 10% internal standards were prepared, along with sample solutions containing 10% wine sample and 10% internal standards, all prepared in triplicates. These solutions were mixed, and filtered through 0.4 µm GC nylon filters into GC vials. The analysis was performed using an Agilent 7890A gas chromatograph (Agilent Technologies, USA) with an injector temperature of 120°C, an oven temperature of 50°C, and a flame ionization detector temperature of 200°C. The procedure used a split ratio of 50:1 and an injection volume of 0.4 µL, following ISO 7609:1985 and SLS 1619:201 standards for alcohol analysis in wine.

#### 2.3.3 Fermentation kinetics model

Alcohol levels, residual sugar, and biomass of each strain were monitored during fermentation (0-14 days). The dilution series was prepared for each wine sample and the pour plate was carried out on WLN agar (Wallerstein Laboratory Nutrient agar, HiMedia, India). The colony count was taken after 24 h of incubation at 28°C. Here, colony counts were taken from the early, middle, and later stages of fermentation of every wine sample (Wang et al., 2022b) to analyze their growth kinetics. For the evaluation of fermentation kinetics, the growth rate constant (r) of yeasts was calculated by using the derived Equation 1 (Zhang et al., 2006).


(1) $${\text{C}}_{\text{t}}={{\text{C}}_{0}\text{e}}^{-\text{rt}}$$

The reaction demonstrated an exponential decrease of residual sugar concentration as a function of time, where C_t_ is the residual sugar concentration at time t, C_0_ is the initial residual sugar concentration (i.e., at time t = 0), and r is the specific rate constant. The next useful sign of the fermentation reaction rate is the time taken for residual sugar concentration to drop to half its initial concentration (half-life, t_1/2_) Zhang et al., 2006). In the case of a first-order reaction, the time taken for the reduction of residual sugar concentration from N_0_ to 1/2 N_0_ is given by Equation 2.


(2) $$\text{N }\left(\text{t}\right)\;={\text{ N}}_{0}\;\left(1/2\right){\;}^{\text{t}/\text{t }(\text{half})}$$

Furthermore, the fermentation kinetics of selected yeast strains were analyzed by using the following Michaelis-Menten Equation 3.


(3) $${\text{V}}_{0}=(\text{Vmax }\left[\text{S}\right]/\left(\text{Km}+\left[\text{S}\right]\right)$$

Where V_0_ is the initial reaction velocity, [S] is the concentration of substrate, Vmax is the maximum reaction velocity and Km is the Michaelis constant (Palma et al., 2012). Here substrate concentration was measured for all selected wine samples during the fermentation period and the graph was plotted showing the reaction rate against the substrate concentration. The substrate concentration was measured as described previously in Section 2.3.1. According to the kinetics the Michaelis constant (Km) is given by the substrate concentration where the reaction rate is half of the maximum value (Vmax). Then R^2^ value of every strain was analyzed to study the goodness of fit of the data to the Michaelis Menten equation.

### 2.4 Determination of pH and acid content

After fermentation (on the 14^th^ day), wine samples from each strain were checked for pH using a pH meter (Hanna Instruments HI 83141, USA) and subjected to High Performance Liquid Chromatography (HPLC) analysis to quantify tartaric acid, malic acid, acetic acid, and lactic acid in the fermented grape wine. For this, organic acids analysis, an Agilent 1260 Infinity HPLC with a quaternary gradient pump, diode array detector (UV-210 nm), and refractive index (RI) detector were used. Chromatographic separation was performed with a Phenomenex Rezex ROA Organic Acid H⁺ 8% (7.8 × 300 mm, 5 μm) analytical column and a guard column (4 × 3 mm). The mobile phase was 0.005 N H_2_SO_4_, with a flow rate of 0.6 mL min^-1^, a total run time of 18 minutes, and an injection volume of 10 µL. The column compartment temperature was 40°C (García-Beneytez et al., 2003).

### 2.5 Fourier Transform Infrared (FTIR) analysis

Wine samples produced by selected strains were analyzed using FTIR (Nicolet iS10 spectrophotometer, Thermo Fisher Scientific, USA) within the range of 4000 to 500 cm⁻¹. KBr pellets with wine samples were prepared and analyzed in transmission mode (Basalekou et al., 2020). The data was processed using OMNIC 7.3 software (Thermo Fisher Scientific Inc.).

### 2.6 E-Nose analysis

The olfactory properties of wine produced by selected yeast strains were analyzed using an E-Nose (AIRSENSE Analytics, PEN3.5), which consists of 10 sensors (**Table 1**). Volatile compounds were detected in graphical format by a 10-fold array of thick film metal oxide gas sensors. The E-Nose underwent a 10-minute pre-warming before each test. A standardized run schedule was employed for all samples, following a two-stage run cycle. The sensors required 100 seconds of sampling run time to attain a stable value. The duration for sensor cleaning before every analysis was set to 100 seconds. The volatile gas samples from the headspace of sealed vials were pumped over the sensors at a flow rate of 200 mL min^-1^, and the sample run analysis was carried out using Winmuster software (AIRSENSE ANALYTICS GmbH, Schwerin, Germany) Sun et al., 2018).

**Table 1:** Sensor sensitivity for the PEN 3.5 sensor array.

Sensor number	Sensor name	Sensor sensitive compounds
1	R(1)	Aromatic organic compounds
2	R(2)	Very sensitive, broad range sensitivity, reacts to NO_2_
3	R(3)	Ammonia, utilized as a sensor for aromatic compounds
4	R(4)	Significantly identify hydrogen gas
5	R(5)	Alkanes, aromatic compounds, and nonpolar organic compounds
6	R(6)	Responsive to methane. A broad range of organic compounds detected
7	R(7)	Recognize inorganic sulfur compounds, e.g. H_2_S. Responsive to several terpenes and sulfur-containing organic compounds
8	R(8)	Recognize alcohol, partially responsive to aromatic compounds, broad range
9	R(9)	Aromatic compounds, inorganic sulfur and organic compounds
10	R(10)	Reacts to high levels (>100 mg/kg) of methane and aliphatic organic compounds

### 2.7 Headspace Solid Phase Microextraction (HS/SPME) - Gas Chromatography-Mass Spectrometry (GC-MS) analysis

The volatile profile of wine samples produced by non-*Saccharomyces* yeast was determined using the SPME-GC-MS method. The headspace SPME sampling conditions were as follows: Initially, 10 mL of the liquid sample, along with 3 g of NaCl, were mixed and carefully transferred into a 20 mL headspace vial. This vial was equipped with a Teflon-lined septum and sealed using an aluminum crimp seal. The contents in the vial were subjected to magnetic stirring for 5 minutes at 60°C. Subsequently, a fiber (DVB/C-WR/PDMS - 50/30 µm, 10 mm, Supelco, Bellefonte, PA, USA) was inserted into the headspace region of the GC-MS (Agilent Technologies 7890A GC, USA) and allowed to remain there for 45 minutes, with a constant fiber length. Desorption of volatile compounds occurred in the injector of the gas chromatograph, operating in spitless mode, at a temperature of 240°C. Before each analysis, the fiber was exposed to the injection port for 5 minutes to remove any volatile contaminants that might be present. The helium flow rate was maintained at 0.8 mL/min during the experiment. The initial oven temperature was set at 40°C for 1 minute, followed by a ramp of 15°C min^-1^ to 140°C, and then a further increase to 260°C for 1.5 minutes. The spectrometer operated in electron impact mode (EI) at 70 eV and a temperature of 240°C for 3 minutes (Wang et al., 2022a).

### 2.8 Sensory evaluation of grape wines

The wine samples were evaluated by quantitative descriptive sensory analysis using a scale from 0 to 9 (0 = very low, 9 = very high) Niu et al., 2011). A group of 11 trained assessors, 5 females and 6 males aged from 25 to 40 involved in the sensory analysis of wine samples. The sensory analysis was carried out according to the reference protocol of Niu et al. (2011). The volatile and visual attributes were evaluated. The volatile profile included fruity, floral, herbaceous, alcoholic, solvent, and phenolic attributes, and the visual profile included color and clarity. The sensory evaluation was carried out at a controlled room temperature between 20-25 °C.

### 2.9 Statistical analysis

Statistical analysis was carried out by using SPSS 20.0 (IBM, US). Tukey’s one-way ANOVA was used to analyze physiochemical and fermentation kinetics parameters to determine the significant differences at a confidence level of 0.05. Data were interpreted as mean ± standard. The Principal Component Analysis (PCA) and heat map graphing were performed using OriginPro 2022 software.

## 3 Results

Twenty-eight non-*Saccharomyces* yeast strains were isolated from grape skin and from different wine fermentation stages (early, middle, and late) of must samples collected from various areas in Sri Lanka. The best performing isolates were identified as *Hanseniaspora opuntiae* J1Y-T1 (OP143841), *H*. *guilliermondii* Y5P-T5 (OP924274), *H*. *uvarum* JF3-T1N (PQ169565), *Pichia kudriavzevii* Y8P-T8 (OP924553), and *Starmerella bacillaris* WMP4-T4 (OP890585) according to their glucose fermenting ability,alcohol production ability and survival ability in ethanol stress (**Supplementary Table 1**).

### 3.1 Yeast population dynamics, substrate utilization and fermentation kinetics

**Figure 1:**
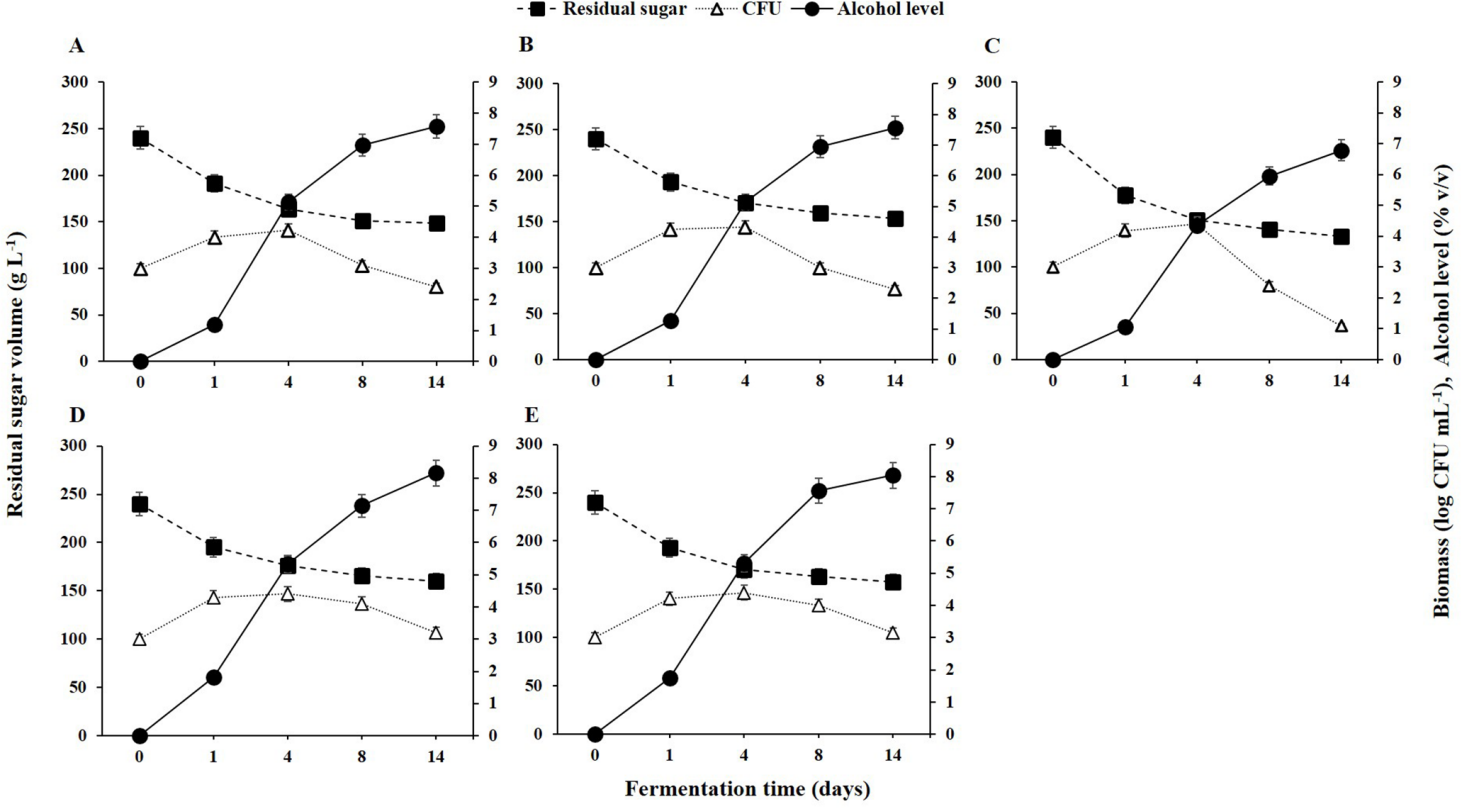
Yeast population dynamics, residual sugar, and alcohol levels during fermentation from 0 to 14 days for different non-*Saccharomyces* yeast strains: **(A)** *H. opuntiae* J1Y-T1; **(B)** *P. kudriavzevii* Y8P-T8; **(C)** *H. guilliermondii* Y5P-T5; **(D)** *H. uvarum* JF3-T1N; and **(E)** *S. bacillaris* WMP4-T4.

As depicted in **Figure 1**, the initial grape juice had a total sugar content of 237.92 ± 3.52 g L⁻¹ and ethanol levels below 0.01 g L⁻¹. During fermentation, residual sugar levels decreased while alcohol levels increased. The growth dynamics of the selected yeast strains on the first day indicated that all five strains entered the exponential growth phase, rapidly utilizing the available sugar.

Despite the efficient initial sugar utilization and rapid growth of *H. guilliermondii* Y5P-T5, its biomass declined after the fourth day due to poor alcohol tolerance (**Figure 1**), with alcohol levels reaching 5% (v/v) by that time. Between days 4 and 14, alcohol levels increased to 8%, but *H. guilliermondii* Y5P-T5 remained the least efficient alcohol producer. In contrast, *H. uvarum* JF3-T1N and *S. bacillaris* WMP4-T4 maintained their populations in high-alcohol conditions, sustaining a biomass of 10³ CFU mL⁻¹.

During the initial fermentation period (days 1-4), all strains exhibited elevated biomass levels, reaching approximately 10⁴ CFU mL⁻¹ (**Figure 1**). However, as fermentation progressed (days 5-8), biomass levels declined due to alcoholic stress. Notably, *H. uvarum* JF3-T1N and *S. bacillaris* WMP4-T4 showed a slower decline compared to the other strains. A significant reduction in sugar levels occurred during the first four days, after which the rates of sugar consumption and alcohol production decreased as colony counts dropped.

By the 14^th^ day of fermentation, the residual sugar levels (g L⁻¹) were as follows: *H. uvarum* JF3-T1N (162.37 ± 2.48) > *S. bacillaris* WMP4-T4 (161.54 ± 3.04) > *P. kudriavzevii* Y8P-T8 (152.16 ± 3.25) > *H. opuntiae* J1Y-T1 (150.34 ± 2.95) > *H. guilliermondii* Y5P-T5 (135.68 ± 2.76). These results indicate that *H. guilliermondii* Y5P-T5 consumed significantly more sugar than the other strains. Similarly, alcohol levels on the 14th day (% v/v) showed the following order: *H. uvarum* JF3-T1N (8.16 ± 0.05) > *S. bacillaris* WMP4-T4 (8.04 ± 0.04) > *P. kudriavzevii* Y8P-T8 (7.57 ± 0.08) > *H. opuntiae* J1Y-T1 (7.57 ± 0.04) > *H. guilliermondii* Y5P-T5 (6.78 ± 0.07), highlighting significant variations in alcohol production among the strains.

Fermentation kinetic studies (**Table 2**) further revealed that *H. guilliermondii* Y5P-T5 exhibited the shortest half-life (2.29 ± 0.03 days) and the highest growth rate (0.302 ± 0.001 day⁻¹), whereas *H. uvarum* JF3-T1N showed the longest half-life (3.34 ± 0.03 days) and the lowest growth rate (0.207 ± 0.001 day⁻¹). No significant differences (p > 0.05) were observed in the half-life and growth rates of *P. kudriavzevii* Y8P-T8 and *S. bacillaris* WMP4-T4.

**Table 2:** GenBank accession numbers and fermentation kinetic parameters of selected non-*Saccharomyces* yeast strains.

Accession number	Yeast species	Half-life (days)	Growth rate (day^-1^)	Vmax (mol L^-1^ day^-1^)	Km (mol L^-1^)	R^2^
OP143841	*Hanseniaspora opuntiae* J1Y-T1	3.051 ± 0.042^c^	0.227 ± 0.001^c^	0.272 ± 0.001^b^	0.993 ± 0.006^c^	0.968 ± 0.000^f^
OP924553	*Pichia kudriavzevii* Y8P-T8	3.193 ± 0.031^d^	0.217 ± 0.001^b^	0.260 ± 0.001^a^	1.003 ± 0.006^cd^	0.923 ± 0.000^d^
OP924274	*Hanseniaspora guilliermondii* Y5P-T5	2.287 ± 0.030^a^	0.302 ± 0.001^e^	0.347 ± 0.001^d^	0.903 ± 0.006^a^	0.920 ± 0.000^c^
PQ169565	*Hanseniaspora uvarum* JF3-T1N	3.341 ± 0.031^e^	0.207 ± 0.001^a^	0.253 ± 0.006^a^	1.020 ± 0.010^d^	0.880 ± 0.000^a^
OP890585	*Starmerella bacillaris* WMP4-T4	3.189 ± 0.022^d^	0.217 ± 0.001^b^	0.257 ± 0.006^a^	0.993 ± 0.006^c^	0.953 ± 0.000^e^

Data are expressed as the means of three samples ± standard deviations. Different letters (a, b, c, d, e, and f) within each column are significantly different (Turkey’s in one way ANOVA; *p* < 0.05)

Kinetic results (**Table 2**) confirmed that *H. guilliermondii* Y5P-T5 had the highest growth rate and Vmax, followed by *H. opuntiae* J1Y-T1. *H. guilliermondii* Y5P-T5 also exhibited the shortest half-life and the lowest Km value. The R² values obtained for all strains showed a strong correlation with the Michaelis-Menten model, with *H. opuntiae* J1Y-T1 and *S. bacillaris* WMP4-T4 showing the best fit, while other strains exhibited slight deviations from this model. In summary, *H. guilliermondii* Y5P-T5 exhibited a significantly higher growth rate during the initial four days of fermentation but struggled under high alcohol conditions. In contrast, *H. uvarum* JF3-T1N and *S. bacillaris* WMP4-T4 maintained their populations and continued alcohol production during the later stages of fermentation.

### 3.2 Acid profile and functional group analysis in fermented wine

**Figure 2:**
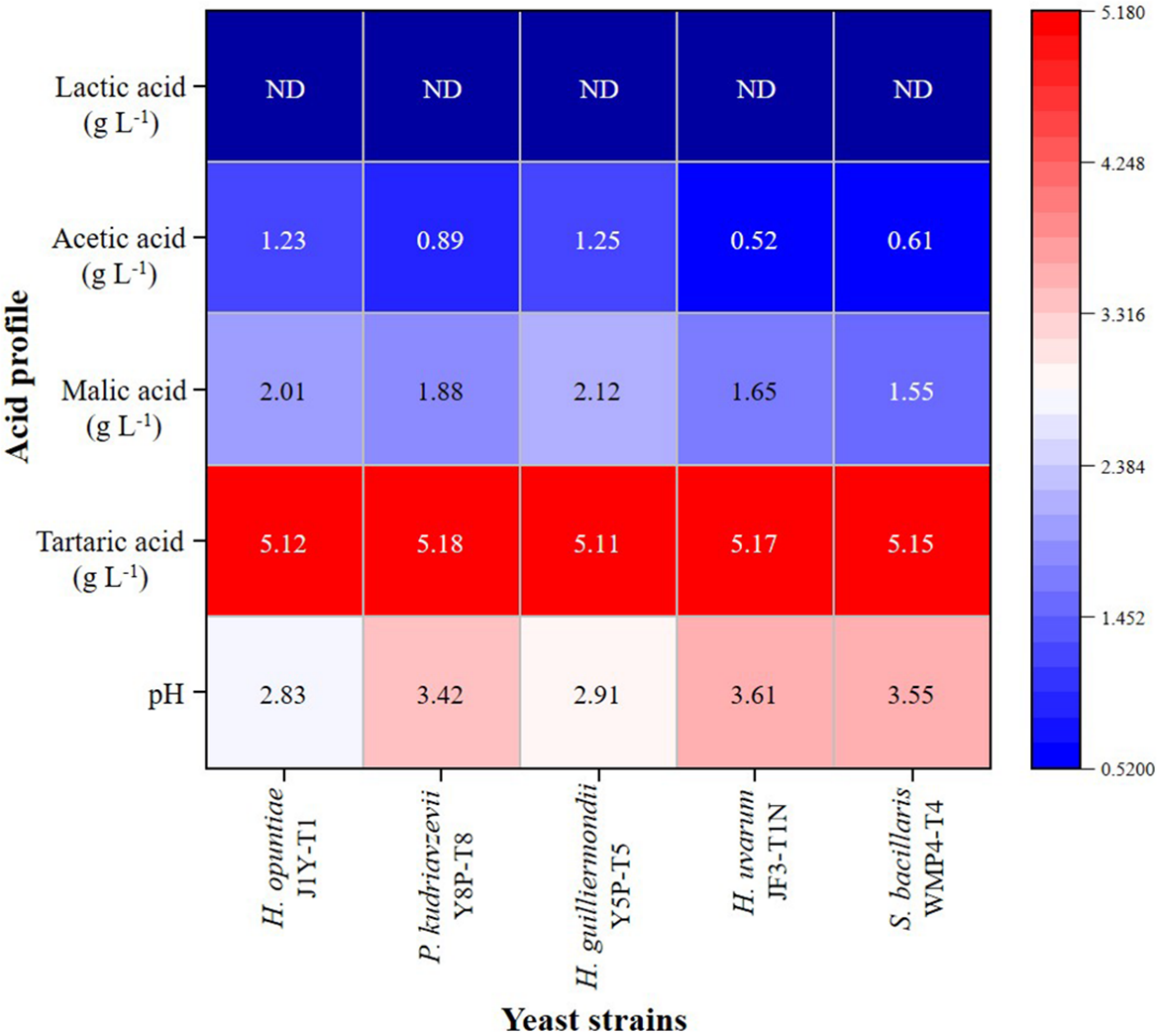
Acid profile heat map of wine samples produced by selected non-*Saccharomyces* yeast strains.

Based on the analysis, the initial grape juice had a pH of 3.89 ± 0.02 and contained tartaric acid (5.21 ± 0.18 g L⁻¹), malic acid (2.24 ± 0.80 g L⁻¹), and acetic acid at levels below 0.01 g L⁻¹. The HPLC results from the fermented wine samples revealed the presence of significant amounts of acetic acid, tartaric acid, and malic acid. As shown in **Figure 2**, the tartaric acid levels in the wine produced by each strain remained relatively consistent with the initial grape juice values. However, malic acid levels showed a significant reduction (p > 0.05) compared to the initial values, particularly in wines fermented with *H. uvarum* JF3-T1N and *S. bacillaris* WMP4-T4, which exhibited the greatest malic acid reductions.

None of the five yeast strains were able to produce lactic acid. Notably, *H. opuntiae* J1Y-T1 and *H. guilliermondii* Y5P-T5 produced significantly higher amounts of acetic acid (greater than 2.0 g L⁻¹) compared to the other strains, while *H. uvarum* JF3-T1N exhibited the lowest acetic acid production. Wines fermented with *H. opuntiae* J1Y-T1 and *H. guilliermondii* Y5P-T5 also displayed elevated levels of fixed acids, which corresponded with their lower pH values (below 3). These lower pH levels contributed to the wines’ overall acidity.

**Figure 3:**
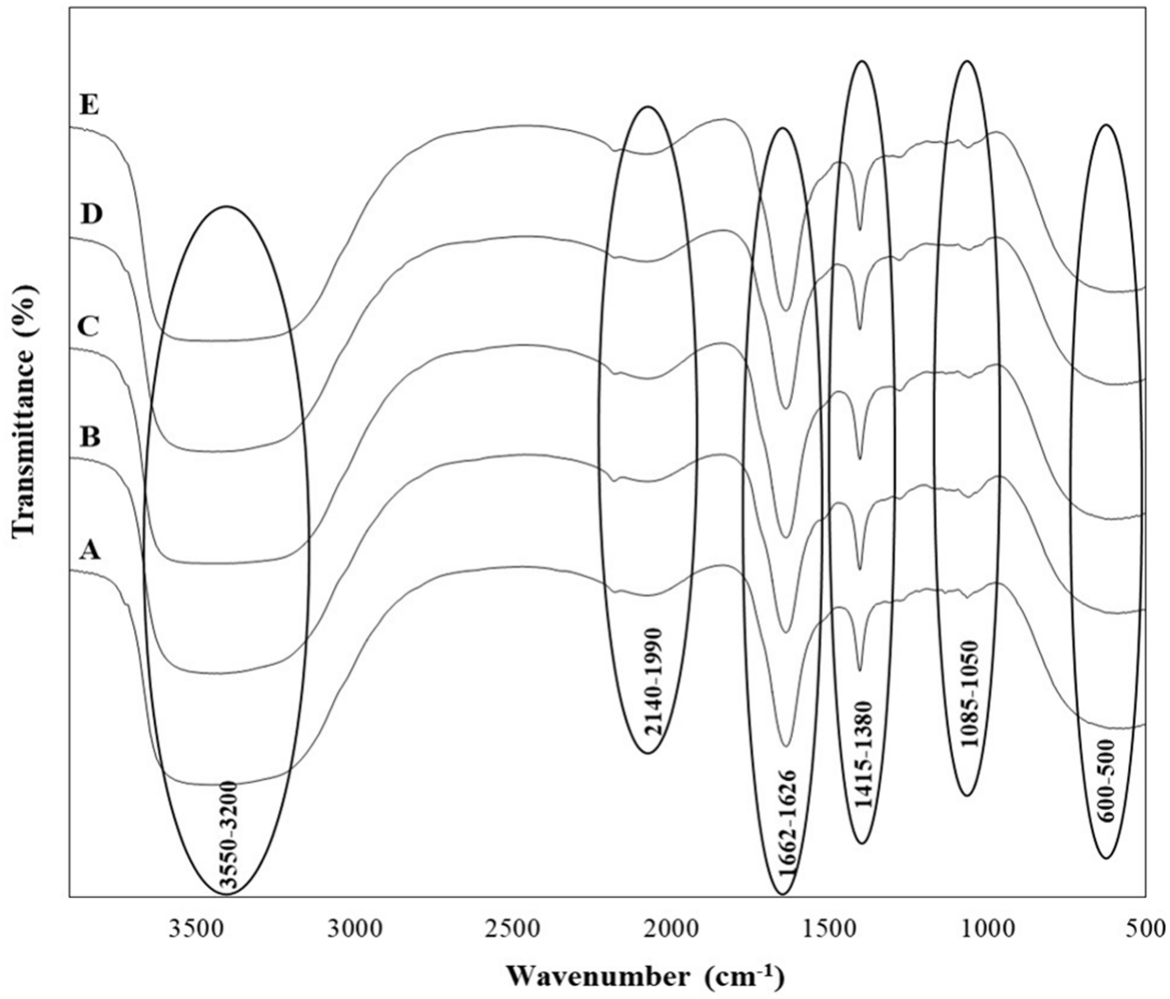
FTIR spectra of wine samples produced by selected non-Saccharomyces yeast strains **(A)** *H. opuntiae* J1Y-T1; **(B)** *P. kudriavzevii *Y8P-T8; **(C)** *H. guilliermondii* Y5P-T5; **(D)** *H. uvarum* JF3-T1N; and **(E)** *S. bacillaris* WMP4-T4.

In addition to the organic acids, alcohols, and sugars identified in the wine samples, their presence was tentatively confirmed by Fourier-transform infrared (FTIR) spectroscopy, as indicated by the functional bonds observed in the spectra (**Figure 3**). Broad O-H stretching from acids and alcohols, along with C-H stretching in hydrocarbons, was detected in the 3200-3550 cm⁻¹ range. Stretching vibrations from -OH, -CH₃, -CH₂, and -CH groups were also observed in the 1626-1662 cm⁻¹ range, while the 1415-1380 cm⁻¹ range exhibited stretching vibrations from -C=O, -C=C, -CH₂, and -CH groups, characteristic of aldehydes, acids, proteins, and esters. The 1050-1085 cm⁻¹ range corresponded to C-O and O-H stretching from sugars and organic acids, and the presence of sulfur compounds was identified in the 600-500 cm⁻¹ range. Phenolic groups were noted around the 1500-1400 cm⁻¹ region, and the 1800-1000 cm⁻¹ range included deformations and stretching vibrations of C-OH, CH₃, CH₂, C=C, and C≡N, indicating the presence of phenols, alcohols, aldehydes, acids, sugars, and amino acids in the wines. The amide I and II regions, which typically range between 1600 and 1700 cm⁻¹, were also observed.

All five wine samples produced by the isolated strains showed nearly identical FTIR spectra, indicating similar chemical compositions across the different fermentation products.

### 3.3 Organoleptic properties of wine produced by isolated non-*Saccharomyces* yeast strains

**Figure 4:**
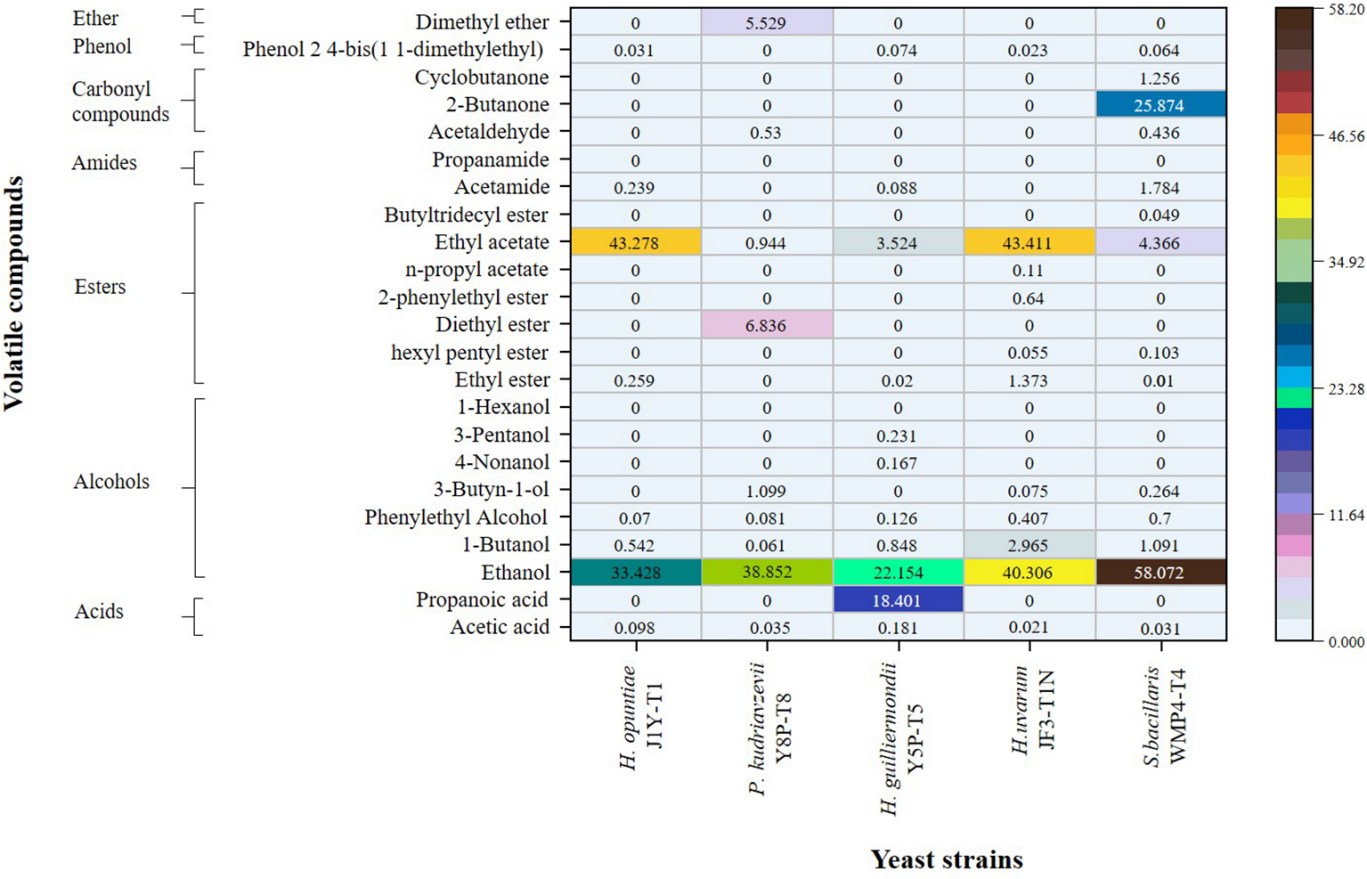
Comparison of the volatile profiles of wine samples produced by selected non-*Saccharomyces* yeast strains through SPME GC/MS analysis.

The quantitative values for the volatile profile of wine samples produced by non-Saccharomyces yeast strains from the Israel blue grapes (*Vitis vinifera* L.) variety are presented as relative peak area percentages in **Figure 4**. Significant differences were observed between the strains (p > 0.05). Notably, *S. bacillaris* WMP4-T4 exhibited the highest level of alcoholic flavor, followed by *H. uvarum* JF3-T1N, while *H. guilliermondii* Y5P-T5 showed the lowest alcohol percentage. This pattern aligns with the alcohol levels observed in **Figure 1**.

In addition to ethanol, trace amounts of volatile compounds, including 1-butanol, phenylethyl alcohol, 3-butyne-1-ol, 4-nonanol, and 3-pentanol, were detected. Among these, *H. guilliermondii* Y5P-T5 was the only strain capable of producing 4-nonanol and 3-pentanol, while *H. guilliermondii* Y5P-T5, *H. uvarum* JF3-T1N, and *S. bacillaris* WMP4-T4 produced low amounts of 3-butyne-1-ol.

Regarding ester production, *H. opuntiae* J1Y-T1 and *H. uvarum* JF3-T1N generated higher amounts of ethyl acetate (43%). *H. uvarum* JF3-T1N produced the greatest variety and total amount of esters, including ethyl ester, hexyl pentyl ester, 2-phenylethyl ester, n-propyl acetate, and ethyl acetate.

In terms of carbonyl compounds, *S. bacillaris* WMP4-T4 was the only strain that produced 2-butanone (25.874 ± 0.6165), along with small amounts of cyclobutanone (1.2558 ± 0.0417) and acetaldehyde (0.4625 ± 0.0374). *P. kudriavzevii* Y8P-T8 also produced a small amount of acetaldehyde (0.5300 ± 0.0424), while the other strains did not produce any carbonyl compounds.

When comparing volatile acidity, acetic acid was present in higher percentages among the strains. The acetic acid levels decreased in the following order: *H. guilliermondii* Y5P-T5 (0.1812 ± 0.0325) > *H. opuntiae* J1Y-T1 (0.0981 ± 0.0134) > *P. kudriavzevii* Y8P-T8 (0.0352 ± 0.0241) > *S. bacillaris* WMP4-T4 (0.0310 ± 0.0388) > *H. uvarum* JF3-T1N (0.0213 ± 0.0134). This trend correlates with the HPLC results for acetic acid presented in **Figure 2**. Additionally, *H. guilliermondii* Y5P-T5 was the only strain to produce a significant amount of propionic acid (18.4010 ± 0.3698).

**Figure 5:**
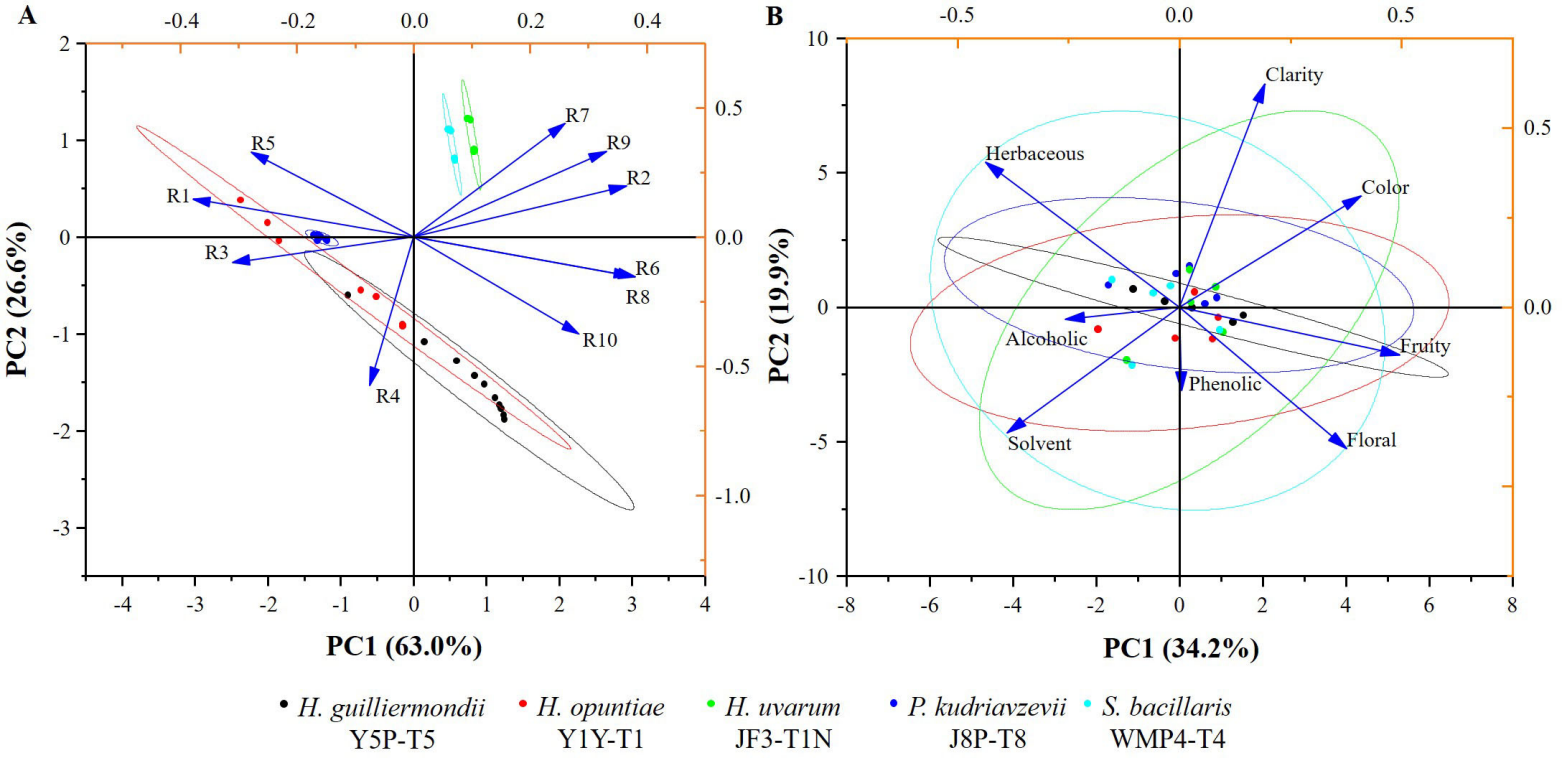
The biplots illustrating the PCA graphs **(A)** for typical E-nose sensory values (R1-R10); **(B)** for sensory attributes of wine samples produced by selected non- *Saccharomyces* yeast strains.

In addition, the E-nose was also used to analyze the volatile profile of produced wine. The wine samples produced by selected strains exhibited similar patterns with minor variations (**Figure 5**). Key sensors such as the broad-range sensor (R2), broad-methane sensor (R6), terpenes and inorganic sulfur sensor (R7), broad-alcohol sensor (R8), and aromatic compounds sensor (R9) showed significant responses.

E-nose and sensory analysis data were utilized for principal component analysis (PCA) to enhance discrimination accuracy by reducing the dimensionality of the data (**Figure 5**). According to **Figure 5A**, the first two principal component scores for the E-nose data, PC1 and PC2, accounted for 63.0% and 26.6% of the variance, respectively, displaying a total variance of about 89.6%. Wine produced by *H. uvarum* JF3-T1N and *S. bacillaris* WMP4-T4 showed positive PC1 and PC2 scores (**Figure 5A**) and were characterized by higher responses of the R2, R6, R7, R8, and R9 sensors compared to other sensors. *H. opuntiae* J1Y-T1*, H. guilliermondii* Y5P-T5, and *P. kudriavzevii* Y8P-T8 showed both negative and positive scores for PC1 and PC2. Notably, the E-nose profiles of *H. opuntiae* J1Y-T1 and *H. guilliermondii* Y5P-T5 overlapped, and they highly correlated with the R1, R3, R4, R5, R6, and R8 sensors. All strains showed a significant response to sensor R8. Additionally*, H. uvarum* JF3-T1N, and *S. bacillaris* WMP4-T4 showed a significant response to R7 during E-nose analysis.

Results of the sensory evaluation are presented in **Figure 5B**. The first two principal component scores of PCA for the sensory evaluation, PC1 and PC2, accounted for 34.2% and 19.9% of the variance, respectively, displaying a total variance of about 54.1%. All strain profiles overlapped around the central value of PC1 and PC2, with slight variations. *S. bacillaris* WMP4-T4 showed balanced values for all the sensory attributes but specifically correlated with herbaceous, solvent, fruity, and floral sensory attributes of wine.

*H. uvarum* JF3-T1N showed lower scores than *S. bacillaris* WMP4-T4 for the herbaceous attribute. Additionally, *H. uvarum* JF3-T1N correlated more with color, and clarity attributes of wines than *S. bacillaris* WMP4-T4. *H. guilliermondii* Y5P-T5 showed a very narrow range of results, mostly herbaceous, fruity, and alcoholic attributes. *H. opuntiae* J1Y-T1 and *P. kudriavzevii* Y8P-T8 leaned more toward alcoholic, solvent, fruity, and herbaceous attributes.

## 4 Discussion

This study aims to isolate efficient wild-type non-*Saccharomyces* yeasts from specific geographical regions of Sri Lanka using Israel blue grapes (*Vitis vinifera* L.) and to correlate their microbial fingerprints to the sensorial properties of the produced wine. *Hanseniaspora opuntiae*, *H*. *guilliermondii*, *H*. *uvarum*, *Pichia kudriavzevii*, and *Starmerella bacillaris* were also identified from previous studies confirming their abundance in grape wine (Tofalo et al., 2011; Cordero-Bueso et al., 2013; Raymond et al., 2017). According to Kalopesa et al. (2023), the total sugar and ethanol content of the initial grape juice were at preferable levels for wine fermentation. After the first day of fermentation, the rapid consumption of sugars by the yeasts resulted in a decrease in residual sugars and an increase in alcohol levels (Renouf et al., 2007). Non-*Saccharomyces* yeasts typically used in sequential wine fermentation can produce alcohol levels ranging from 7 to 14 g L^-1^, and generally show less tolerance to high alcohol concentrations (Vilela, 2019). Specifically, in this study, *H. opuntiae* J1Y-T1*, H. guilliermondii* Y5P-T5, and *P. kudriavzevii* Y8P-T8 showed poor alcohol tolerance beyond 5% (v/v). In contrast, *H. uvarum* JF3-T1N and *S. bacillaris* WMP4-T4 showed remarkable alcohol tolerance, maintaining a significantly higher biomass level in the late fermentation stage. Microorganisms that survived during the late fermentation stage significantly influence the sensory properties of wine (Renouf et al., 2007; Hranilovic et al., 2018).

Generally, in wine fermentation, the amount of glucose in the must determines the fermentation rate (Nissen et al., 2004). At low sugar concentrations, yeast growth is controlled by sugar availability, and the fermentation rate shows a positive correlation with sugar concentration (Palma et al., 2012). At high sugar concentrations, the fermentation rate depends on the yeast’s ability to consume sugar until it reaches the maximum rate (Vmax) Galaction et al., 2010). The higher Vmax shown by *H. guilliermondii* Y5P-T5 and *H. opuntiae* J1Y-T1 indicates their efficient substrate utilization. Further, the Km value indicates the affinity of yeast enzymes for sugar during fermentation, with a lower value suggesting they can effectively consume sugar even at lower concentrations (Robinson, 2015). Here, *H. guilliermondii* Y5P-T5 exhibited a lower Km value, indicating higher sugar affinity, which correlated with its highest growth rate (**Table 2**). Consequently, wines from *H. guilliermondii* Y5P-T5 efficiently utilized sugar during fermentation, which tended to reduce the residual sugars and could affect the sensory properties of wine.

The maximum R² values obtained for *H. opuntiae* J1Y-T1 and *S. bacillaris* WMP4-T4 indicated the best fit to the Michaelis-Menten model, attributed to their high alcohol tolerance and survival. The deviation observed in other strains was due to alcohol toxicity during extended fermentation. However, R² values alone cannot fully determine the fermenting ability of yeast strains, although they can be useful for monitoring and optimizing the fermentation process.

In terms of acid profile tartaric acid, followed by malic acid, is primarily found in grapes and constitutes the most abundant fixed acid in grape wines (Izquierdo-Llopart et al., 2020). L-Tartaric acid is resistant to microbial degradation. Hence, the initial tartaric acid values remained almost consistent, but a slight reduction occurred possibly due to precipitation as calcium or potassium tartrate during the fermentation process (Dutraive et al., 2019; Cioch-Skoneczny et al., 2021). In this study, significant reductions in malic acid concentration were observed for *P. kudriavzevii* Y8P-T8*, H. uvarum* JF3-T1N, and *S. bacillaris* WMP4-T4 after fermentation, which may be due to their partial metabolism of malic acid (Vilela, 2019; Cioch-Skoneczny et al., 2021). Further, acetic acid produced during fermentation plays a crucial role in determining the quality of wine as it affects the total acidity and volatile acidity of wine. The typical threshold of acetic acid in wine is 0.8 g L^-1^; beyond which the wine may develop undesired vinegar characteristics (Benito et al., 2019; Vilela, 2019). Therefore, wines produced by *H. opuntiae* J1Y-T1 and *H. guilliermondii* Y5P-T5 were mostly considered unpleasant. Acetic acid fermenting microorganisms involved in wine fermentation can oxidize alcohol into acetic acid (Mas et al., 2014), which causes a reduction in alcohol level. Hence, *H. opuntiae* J1Y-T1 and *H. guilliermondii* Y5P-T5 showed lower alcohol levels. All five strains identified in this study were unable to produce lactic acid as they do not engage in malolactic fermentation (Virdis et al., 2021). According to Kalopesa et al. (2023), the obtained results in this study for tartaric acid, malic acid, and acetic acid fall within the acceptable range of wine.

The preferable pH range for wine is 3-4 (Morata et al., 2021). But *H. opuntiae* J1Y-T1 and *H. guilliermondii* Y5P-T5 demonstrated a lesser pH level making them more acidic than other wine samples. However, pH levels above 4 typically increase susceptibility to spoilage (Morata et al., 2021). *H. uvarum* JF3-T1N and *S. bacillaris* WMP4-T4 showed higher pH and alcohol levels, with lower acetic acid levels compared to other strains, providing preferable organoleptic qualities to the final product. Previous studies indicated that using *S. bacillaris* positively affects wine characteristics by reducing excess ethanol and acetic acid levels (Nisiotou et al., 2018; Vilela, 2019; Li et al., 2023). The level of total sugar, ethanol, pH, and total acids in wines from each strain obtained in this study aligned with the People’s Republic of China national standard (GB/T15038-2006), confirming the required quality of the wine.

In the FTIR analysis, all the wine samples from the five different yeasts showed similar functional groups (**Figure 3**), which align with the previous studies conducted by Zhang et al. (2010); Budziak-Wieczorek et al. (2023), and Teixeira dos Santos et al. (2024). This includes functional groups related to carbonyl compounds, esters, acids, alcohols, and phenols in the wine (**Figure 3**). The functional groups observed related to the acids were due to the presence of tartaric, malic and acetic acids, while the bonds for alcohol were mainly due to ethanol. The peaks related to amide groups served as indicators of wine type and sweetness, while the 1580-950 cm⁻¹ range, rich in various compounds, indicated wine quality (Budziak-Wieczorek et al., 2023). Some peaks presented in other studies were not identified here, likely due to differences in grape types, yeast starter cultures, and winemaking and aging techniques.

The presence of carbonyl compounds, esters, acetates, terpenes, acids, alcohols, and phenols reflects the organoleptic properties in wine (Gómez-Míguez et al., 2007; Borren and Tian, 2020). Higher amounts of volatile acids produced by *H. guilliermondii* Y5P-T5 also caused a lower pH value in the wine, as previously described. The higher amount and varieties of esters produced by *H. uvarum* JF3-T1N could positively affect the aroma profile, as esters contribute to highly preferable fruity flavor and floral aroma profile (Benito et al., 2019; Dutraive et al., 2019; Borren and Tian, 2020). Acetaldehyde, one of the main carbonyl compounds observed in the produced wine, was an intermediate in acetic acid production, corroborating the results of Borren and Tian (2020). Overall, *S. bacillaris* WMP4-T4 and *H. uvarum* JF3-T1N exhibited a higher number of favorable volatile wine compounds compared to other non-*Saccharomyces* yeast strains, making them more preferable.

The sensors in the E-nose provided specific values for different wine samples. The corresponding sensory readings by the E-nose were defined as the ratio between the conductance of the wine sample (G) and the carrier gas or baseline signal (G0) over time (Gardner and Bartlett, 2000; Cao et al., 2020), effectively depicting the specific patterns of the sensor responses to the wine samples. The closely associated or overlapping wine samples in PCA possess almost the same sensory compounds and volatile aromas (Lozano et al., 2005). This indicates a similarity between *H. guilliermondii* Y5P-T5 and *H. opuntiae* J1Y-T1 in aroma profile, as well as a similarity between *H. uvarum* JF3-T1N and *S. bacillaris* WMP4-T4.

The E-nose sensor showed higher values in sensors R7, R8, and R9 for wines produced by *H. uvarum* JF3-T1N and *S. bacillaris* WMP4-T4, indicating a preferable aroma profile in winemaking. The higher response was shown for sensor R8 by all the strains correlated with their alcohol production. Wines produced by *H. opuntiae* J1Y-T1 and *P. kudriavzevii* Y8P-T8 contained higher amounts of terpenes, inorganic sulfur, and aromatic compounds than other sensory compounds. *H. opuntiae* J1Y-T1 showed a high level of broad-methane presence due to the response of sensor R6 during e-nose analysis, contributing to an unpleasant taste in wine, making it less preferable. The response for sensor R9 was significantly higher for *H. uvarum* JF3-T1N and *S. bacillaris* WMP4-T4, correlating with the GC-SPME results as they produced higher amounts of aromatic compounds. Hence, the volatile and other sensory compounds present in the wine samples can be confirmed based on the data produced from GC-MS-SPME and E-nose analyses.

Even though *S. bacillaris* WMP4-T4 and *H. uvarum* JF3-T1N were noted as the best candidates for wine production based on fermentation ability and volatile profile analysis, the wine produced with *H. uvarum* JF3-T1N preferred by panelists in sensory evaluation. Due to its production of more esters, which impart a more pleasant aroma and taste, reduce herbaceous flavor, and enhance color and clarity, *H. uvarum* JF3-T1N is the most preferred. Also, it has an acceptable alcohol percentage in wine (8.16 ± 0.05% v/v), less acetic acid, and contains significant amounts of residual sugars, making it more palatable (**Figures 1**, **2**).

Considering all other analyses and fermentation kinetics, *H. uvarum* JF3-T1N appears to have advantages over other strains, making it preferable to use in coculture with *Saccharomyces* species in wine production. A recent study also revealed that the sequential inoculation of *H. uvarum* with *S. cerevisiae* enhanced the polyphenolic and volatile compounds of the final wine, resulting in pleasant characteristics (Testa et al., 2021).

This study confirmed the role of non- *Saccharomyces* yeasts from Sri Lankan grapes in wine sensory properties. Mixed culture applications of *S*. *cerevisiae* with non-*Saccharomyces* yeasts like *Starmerella bacillaris*, *Pichia* spp., and *Hanseniaspora* spp. could enhance flavor diversity and complexity (Martin et al., 2018; Benito et al., 2019; Dutraive et al., 2019; Vilela, 2019). Therefore, these selected strains can be used in coculture with *S. cerevisiae* to create starter cultures for wine production with favorable characteristics. Furthermore, immobilization techniques can be used to immobilize these cultures in the optimal carrier medium, allowing them to be reused for continuous winemaking process on a large scale (Moreno-García et al., 2018). These approaches will open up new avenues in the winemaking industry.

## 5 Conclusion

Among twenty-eight non-Saccharomyces yeast isolates from *Vitis vinifera* L., five strains—*Hanseniaspora opuntiae* J1Y-T1, *Pichia kudriavzevii* Y8P-T8, *H*. *guilliermondii* Y5P-T5, *H*. *uvarum* JF3-T1N, and *Starmerella bacillaris* WMP4-T4—demonstrated promising potential for fermentation, exhibiting rapid initial sugar consumption and efficient alcohol production. Notably, *H. guilliermondii* Y5P-T5 showed instant sugar utilization and growth but faced challenges with high alcohol concentrations later in fermentation. In contrast, *H. uvarum* JF3-T1N and *S. bacillaris* WMP4-T4 displayed superior alcohol tolerance, maintaining high alcohol levels (~8-10% v/v) and yeast biomass throughout the process. Each strain influenced wine acidity differently, with *H. opuntiae* J1Y-T1 and *H. guilliermondii* Y5P-T5 producing elevated acetic acid levels that negatively impacted wine quality, while *H. uvarum* JF3-T1N and *S. bacillaris* WMP4-T4 maintained lower acetic acid levels and a favorable pH range. The volatile compound profiles varied significantly among the analyzed wine samples, with *H. uvarum* JF3-T1N and *S. bacillaris* WMP4-T4 yielding more desirable aromatic compounds, particularly fruity and floral esters from *H. uvarum*, enhancing the wine’s sensory appeal. Due to its reduced herbaceous taste and enhanced color, *H. uvarum* JF3-T1N emerges as the preferred strain, showing great promise for coculture fermentations with *S*. *cerevisiae* to create wines that reflect Sri Lankan flavors. These findings underscore the critical role of strain selection in optimizing wine quality through controlled fermentation processes.

## Data Availability

The datasets presented in this study can be found in online repositories. The names of the repository/repositories and accession number(s) can be found in the article/**Supplementary Material**.

## References

[B1] Albergaria H. Arneborg N. (2016). Dominance of Saccharomyces cerevisiae in alcoholic fermentation processes: role of physiological fitness and microbial interactions. Appl. Microbiol. Biotechnol. 100, .2035–.2046. doi: 10.1007/s00253-015-7255-0 26728020

[B2] Barata A. Malfeito-Ferreira M. Loureiro V. (2012). The microbial ecology of wine grape berries. Int. J. Food Microbiol. 153, 243–259. doi: 10.1016/j.ijfoodmicro.2011.11.025 22189021

[B3] Barnett J. A. Payne R. W. Yarrow D. (2000). Yeasts- characteristics and identification. New York: Cambridge University Press.

[B4] Basalekou M. Pappas C. Tarantilis P. A. Kallithraka S. (2020). Wine authenticity and traceability with the use of FT-IR. Beverages 6, 30. doi: 10.3390/beverages6020030

[B5] Benito Á. Calderón F. Benito S. (2019). The influence of non-*Saccharomyces* species on wine fermentation quality parameters. Fermentation 5, 54. doi: 10.3390/fermentation5030054

[B6] Berbegal C. Khomenko I. Russo P. Spano G. Fragasso M. Biasioli F.. (2020). PTR-ToF-MS for the online monitoring of alcoholic fermentation in wine: Assessment of VOCs variability associated with different combinations of *Saccharomyces*/non-*Saccharomyces* as a case-study. Fermentation 6, 55. doi: 10.3390/fermentation6020055

[B7] Bokulich N. A. Thorngate J. H. Richardson P. M. Mills D. A. (2014). Microbial biogeography of wine grapes is conditioned by cultivar, vintage, and climate. Proc. Natl. Acad. Sci. 111, E139–E148. doi: 10.1073/pnas.1317377110 24277822 PMC3890796

[B8] Borren E. Tian B. (2020). The important contribution of non-Saccharomyces yeasts to the aroma complexity of wine: A review. Foods 10, 13. doi: 10.3390/foods10010013 33374550 PMC7822458

[B9] Budziak-Wieczorek I. Mašán V. Rząd K. Gładyszewska B. Karcz D. Burg P.. (2023). Evaluation of the quality of selected white and red wines produced from Moravia Region of Czech Republic using physicochemical analysis, FTIR infrared spectroscopy and chemometric techniques. Molecules 28, 6326. doi: 10.3390/molecules28176326 37687155 PMC10489813

[B10] Čadež N. Zupan J. Raspor P. (2010). The effect of fungicides on yeast communities associated with grape berries. FEMS yeast Res. 10, 619–630. doi: 10.1111/j.1567-1364.2010.00635.x 20491940

[B11] Camilo S. Chandra M. Branco P. Malfeito-Ferreira M. (2022). Wine microbial consortium: Seasonal sources and vectors linking vineyard and winery environments. Fermentation 8, 324. doi: 10.3390/fermentation8070324

[B12] Cao Y. Wu Z. Weng P. (2020). Comparison of bayberry fermented wine aroma from different cultivars by GC-MS combined with electronic nose analysis. Food Sci. Nutr. 8, 830–840. doi: 10.1002/fsn3.1343 32148792 PMC7020313

[B13] Capece A. Siesto G. Romaniello R. Lagreca V. M. Pietrafesa R. Calabretti A.. (2013). Assessment of competition in wine fermentation among wild *Saccharomyces cerevisiae* strains isolated from Sangiovese grapes in Tuscany region. LWT-Food Sci. Technol. 54, 485–492. doi: 10.1016/j.lwt.2013.07.001

[B14] Ciani M. Comitini F. (2019). “Yeast ecology of wine production,” in Yeasts in the production of wine. New York: Springer. 1–42. doi: 10.1007/978-1-4939-9782-4_1

[B15] Ciani M. Comitini F. Mannazzu I. Domizio P. (2010). Controlled mixed culture fermentation: a new perspective on the use of non-*Saccharomyces* yeasts in winemaking. FEMS yeast Res. 10, 123–133. doi: 10.1111/j.1567-1364.2009.00579.x 19807789

[B16] Cioch-Skoneczny M. Grabowski M. Satora P. Skoneczny S. Klimczak K. (2021). The use of yeast mixed cultures for deacidification and improvement of the composition of cold climate grape wines. Molecules 26, 2628. doi: 10.3390/molecules26092628 33946291 PMC8125709

[B17] Cocolin L. Bisson L. F. Mills D. A. (2000). Direct profiling of the yeast dynamics in wine fermentations. FEMS Microbiol. Lett. 189, 81–87. doi: 10.1111/j.1574-6968.2000.tb09210.x 10913870

[B18] Combina M. Elía A. Mercado L. Catania C. Ganga A. Martinez C. (2005). Dynamics of indigenous yeast populations during spontaneous fermentation of wines from Mendoza, Argentina. Int. J. Food Microbiol. 99, 237–243. doi: 10.1016/j.ijfoodmicro.2004.08.017 15808358

[B19] Cordero-Bueso G. Esteve-Zarzoso B. Cabellos J. M. Gil-Díaz M. Arroyo T. (2013). Biotechnological potential of non-Saccharomyces yeasts isolated during spontaneous fermentations of Malvar (*Vitis vinifera* cv. L.). Eur. Food Res. Technol. 236, 193–207. doi: 10.1007/s00217-012-1874-9

[B20] De Gioia M. Russo P. De Simone N. Grieco F. Spano G. Capozzi V.. (2022). Interactions among relevant non-saccharomyces, saccharomyces, and lactic acid bacteria species of the wine microbial consortium: towards advances in antagonistic phenomena and biocontrol potential. Appl. Sci. 12, 12760. doi: 10.3390/app122412760

[B21] Duka G. Soldatenco G. O. Taran N. (2024). “The impact of non-saccharomyces yeasts on grape must fermentation: comprehensive study,” in Bulletin of the transilvania university of brasov, series II: forestry, wood industry (Brasov, Romania: Agricultural Food Engineering), 141–152. doi: 10.31926/but.fwiafe.2024.17.66.1.8

[B22] Dutraive O. Benito S. Fritsch S. Beisert B. Patz C. D. Rauhut D. (2019). Effect of sequential inoculation with non-*Saccharomyces* and *Saccharomyces* yeasts on Riesling wine chemical composition. Fermentation 5, 79. doi: 10.3390/fermentation5030079

[B23] Fazio N. A. Russo N. Foti P. Pino A. Caggia C. Randazzo C. L. (2023). Inside current winemaking challenges: Exploiting the potential of conventional and unconventional yeasts. Microorganisms 11, 1338. doi: 10.3390/microorganisms11051338 37317312 PMC10222344

[B24] Fleet G. H. (2003). Yeast interactions and wine flavour. Int. J. Food Microbiol. 86, 11–22. doi: 10.1016/S0168-1605(03)00245-9 12892919

[B25] Fleet G. H. (2008). Wine yeasts for the future. FEMS yeast Res. 8, 979–995. doi: 10.1111/j.1567-1364.2008.00427.x 18793201

[B26] Franco W. Benavides S. Valencia P. Ramírez C. Urtubia A. (2021). Native yeasts and lactic acid bacteria isolated from spontaneous fermentation of seven grape cultivars from the maule region (Chile). Foods 10, 1737. doi: 10.3390/foods10081737 34441515 PMC8391128

[B27] Galaction A. I. Lupăşteanu A. M. Caşcaval D. (2010). Kinetic studies on alcoholic fermentation under substrate inhibition conditions using a bioreactor with stirred bed of immobilized yeast cells. Open Syst. Biol. J. 3, 9–20. doi: 10.2174/1876392801003010009

[B28] García-Beneytez E. Cabello F. Revilla E. (2003). Analysis of grape and wine anthocyanins by HPLC-MS. J. Agric. Food Chem. 51, 5622–5629. doi: 10.1021/jf0302207 12952411

[B29] Gardner J. W. Bartlett P. N. (2000). Electronic noses. Principles applications. Measurement Sci. Technol. 11, 1087–1087. doi: 10.1093/oso/9780198559559.001.0001

[B30] Gómez-Míguez M. J. Gómez-Míguez M. Vicario I. M. Heredia F. J. (2007). Assessment of colour and aroma in white wines vinifications: Effects of grape maturity and soil type. J. Food Eng. 79, 758–764. doi: 10.1016/j.jfoodeng.2006.02.038

[B31] Hanamant R. H. Suresha G. J. Jagadeesh S. L. Kalagudi V. A. (2015). Influence of thermovinification on quality of jamun (Syzygium cumini) wine. Int. J. Food Fermentation Technol. 5, 259–263. doi: 10.5958/2277-9396.2016.00018.0

[B32] Hranilovic A. Li S. Boss P. K. Bindon K. Ristic R. Grbin P. R.. (2018). Chemical and sensory profiling of Shiraz wines co-fermented with commercial non-*Saccharomyces* inocula. Aust. J. Grape Wine Res. 24, 166–180. doi: 10.1111/ajgw.12320

[B33] Izquierdo-Llopart A. Carretero A. Saurina J. (2020). Organic acid profiling by liquid chromatography for the characterization of base vines and sparkling wines. Food Analytical Methods 13, 1852–1866. doi: 10.1007/s12161-020-01808-1

[B34] Kalopesa E. Gkrimpizis T. Samarinas N. Tsakiridis N. L. Zalidis G. C. (2023). Rapid determination of wine grape maturity level from pH, titratable acidity, and sugar content using non-destructive *in situ* infrared spectroscopy and multi-head attention convolutional neural networks. Sensors 23, 9536. doi: 10.3390/s23239536 38067909 PMC10708745

[B35] Kántor A. Mareček J. Ivanišová E. Terentjeva M. Kačániová M. (2017). “Microorganisms of grape berries,” in Proceedings of the Latvian academy of sciences. Section B. Natural, exact, and applied sciences, vol. 71. , 502–508).

[B36] Kurtzman C. P. Robnett C. (1997). Identification of clinically important ascomycetous yeasts based on nucleotide divergence in the 5'end of the large-subunit (26S) ribosomal DNA gene. J. Clin. Microbiol. 35, 1216–1223. doi: 10.1128/jcm.35.5.1216-1223.1997 9114410 PMC232732

[B37] Li R. Liu Y. Zheng J. Xu M. Wang H. Sun C.. (2023). Oenological characteristics of two indigenous *Starmerella bacillaris* strains isolated from Chinese wine regions. Appl. Microbiol. Biotechnol. 107, 3717–3727. doi: 10.1007/s00253-023-12502-7 37097503

[B38] López-Enríquez L. Vila-Crespo J. Rodríguez-Nogales J. M. Fernández-Fernández E. Ruipérez V. (2023). Non-saccharomyces yeasts from organic vineyards as spontaneous fermentation agents. Foods 12, 3644. doi: 10.3390/foods12193644 37835297 PMC10572797

[B39] Lozano J. Santos J. P. Horrillo M. C. (2005). Classification of white wine aromas with an electronic nose. Talanta 67, 610–616. doi: 10.1016/j.talanta.2005.03.015 18970214

[B40] Maicas S. Mateo J. J. (2023). The life of *Saccharomyces* and non-*Saccharomyces* yeasts in drinking wine. Microorganisms 11, 1178. doi: 10.3390/microorganisms11051178 37317152 PMC10224428

[B41] Martin V. Valera M. J. Medina K. Boido E. Carrau F. (2018). Oenological impact of the Hanseniaspora/Kloeckera yeast genus on wines—A review. Fermentation 4, 76. doi: 10.3390/fermentation4030076

[B42] Mas A. Torija M. J. García-Parrilla M. D. C. Troncoso A. M. (2014). Acetic acid bacteria and the production and quality of wine vinegar. Sci. World J. 2014, 394671. doi: 10.1155/2014/394671 PMC3918346 24574887

[B43] Mills D. A. Johannsen E. A. Cocolin L. (2002). Yeast diversity and persistence in botrytis-affected wine fermentations. Appl. Environ. Microbiol. 68, 4884–4893. doi: 10.1128/AEM.68.10.4884-4893.2002 12324335 PMC126389

[B44] Morata A. Loira I. González C. Escott C. (2021). Non-*Saccharomyces* as biotools to control the production of off-flavors in wines. Molecules 26, 4571. doi: 10.3390/molecules26154571 34361722 PMC8348789

[B45] Moreno-García J. García-Martínez T. Mauricio J. C. Moreno J. (2018). Yeast immobilization systems for alcoholic wine fermentations: actual trends and future perspectives. Front. Microbiol. 9, 241. doi: 10.1007/s11947-010-0379-4 29497415 PMC5819314

[B46] Nardi T. (2020). Microbial resources as a tool for enhancing sustainability in winemaking. Microorganisms 8, 507. doi: 10.3390/microorganisms8040507 32252445 PMC7232173

[B47] Nisiotou A. Sgouros G. Mallouchos A. Nisiotis C. S. Michaelidis C. Tassou C.. (2018). The use of indigenous *Saccharomyces cerevisiae* and *Starmerella bacillaris* strains as a tool to create chemical complexity in local wines. Food Res. Int. 111, 498–508. doi: 10.1016/j.foodres.2018.05.035 30007712

[B48] Nissen P. Nielsen D. Arneborg N. (2004). The relative glucose uptake abilities of non-*Saccharomyces* yeasts play a role in their coexistence with *Saccharomyces cerevisiae* in mixed cultures. Appl. Microbiol. Biotechnol. 64, 543–550. doi: 10.1007/s00253-003-1487-0 14689245

[B49] Niu Y. Zhang X. Xiao Z. Song S. Eric K. Jia C.. (2011). Characterization of odor-active compounds of various cherry wines by gas chromatography–mass spectrometry, gas chromatography–olfactometry and their correlation with sensory attributes. J. Chromatogr. B 879, 2287–2293. doi: 10.1016/j.jchromb.2011.06.015 21727038

[B50] Onetto C. A. Ward C. M. Van Den Heuvel S. Hale L. Cuijvers K. Borneman A. R. (2024). Temporal and spatial dynamics within the fungal microbiome of grape fermentation. Environ. Microbiol. 26, e16660. doi: 10.1111/1462-2920.16660 38822592

[B51] Palma M. Madeira S. C. Mendes-Ferreira A. Sá-Correia I. (2012). Impact of assimilable nitrogen availability in glucose uptake kinetics in *Saccharomyces cerevisiae* during alcoholic fermentation. Microbial Cell factories 11, 1–11. doi: 10.1186/1475-2859-11-99 22846176 PMC3503800

[B52] Pinto C. Pinho D. Sousa S. Pinheiro M. Egas C. C. Gomes A. (2014). Unravelling the diversity of grapevine microbiome. PloS One 9, 85622. doi: 10.1371/journal.pone.0085622 PMC3894198 24454903

[B53] Raymond Eder M. L. Reynoso C. Lauret S. C. Rosa A. L. (2017). Isolation and identification of the indigenous yeast population during spontaneous fermentation of Isabella (*Vitis labrusca* L.) grape must. Front. Microbiol. 8. doi: 10.3389/fmicb.2017.00532 PMC5372804 28424672

[B54] Renouf V. Claisse O. Lonvaud-Funel A. (2007). Inventory and monitoring of wine microbial consortia. Appl. Microbiol. Biotechnol. 75, 149–164. doi: 10.1007/s00253-006-0798-3 17235561

[B55] Ribereau-Gayon P. Dubourdieu D. Donèche B. Lonvaud A. (2005). “Handbook of enology: volume 1,” in The microbiology of wine and vinifications (West Sussex, England: John Wiley & Sons, Ltd).

[B56] Robinson P. K. (2015). Enzymes: principles and biotechnological applications. Essays Biochem. 59, 1. doi: 10.1042/bse0590001 26504249 PMC4692135

[B57] Romano P. Braschi G. Siesto G. Patrignani F. Lanciotti R. (2022). Role of yeasts on the sensory component of wines. Foods 11, 1921. doi: 10.3390/foods11131921 35804735 PMC9265420

[B58] Schober D. Wacker M. Schmarr H. G. Fischer U. (2023). Understanding the contribution of co-fermenting non-saccharomyces and saccharomyces yeasts to aroma precursor degradation and formation of sensory profiles in wine using a model system. Fermentation 9, 931. doi: 10.3390/fermentation9110931

[B59] Setati M. E. Jacobson D. Andong U. C. Bauer F. (2012). The vineyard yeast microbiome, a mixed model microbial map. PloS One 7, E52609. doi: 10.1371/journal.pone.0052609 23300721 PMC3530458

[B60] Steensels J. Verstrepen K. J. (2014). Taming wild yeast: potential of conventional and nonconventional yeasts in industrial fermentations. Annu. Rev. Microbiol. 68, 61–80. doi: 10.1146/annurev-micro-091213-113025 24773331

[B61] Sun F. Wu Z. Chen Y. Li J. He S. Bai R. (2018). Analysis of odors from thermally modified bamboo assessed by an electronic nose. Building Environ. 144, 386–391. doi: 10.1016/j.buildenv.2018.08.057

[B62] Taylor M. W. Tsai P. Anfang N. Ross H. A. Goddard M. R. (2014). Pyrosequencing reveals regional differences in fruit-associated fungal communities. Environ. Microbiol. 16, 2848–2858. doi: 10.1111/1462-2920.12456 24650123 PMC4257574

[B63] Teixeira dos Santos C. A. Páscoa R. N. M. J. Pérez-del-Notario N. González-Sáiz J. M. Pizarro C. Lopes J. A. (2024). Application of fourier-transform infrared spectroscopy for the assessment of wine spoilage indicators: A feasibility study. Molecules 29, 1882. doi: 10.3390/molecules29081882 38675701 PMC11054220

[B64] Testa B. Coppola F. Lombardi S. J. Iorizzo M. Letizia F. Di Renzo M.. (2021). Influence of *Hanseniaspora uvarum* AS27 on chemical and sensorial characteristics of aglianico wine. Processes 9, 326. doi: 10.3390/pr9020326

[B65] Tofalo R. Schirone M. Telera G. C. Manetta A. C. Corsetti A. Suzzi G. (2011). Influence of organic viticulture on non-*Saccharomyces* wine yeast populations. Ann. Microbiol. 61, 57–66. doi: 10.1007/s13213-010-0102-8

[B66] Tofalo R. Suzzi G. Perpetuini G. (2021). Discovering the influence of microorganisms on wine color. Front. Microbiol. 12. doi: 10.3389/fmicb.2021.790935 PMC8678073 34925298

[B67] Tufariello M. Fragasso M. Pico J. Panighel A. Castellarin S. D. Flamini R.. (2021). Influence of non-*Saccharomyces* on wine chemistry: A focus on aroma-related compounds. Molecules 26, 644. doi: 10.3390/molecules26030644 33530641 PMC7865429

[B68] Van Leeuwen C. Seguin G. (2006). The concept of terroir in viticulture. J. Wine Res. 17, 1–10. doi: 10.1080/09571260600633135

[B69] Vilela A. (2019). Use of nonconventional yeasts for modulating wine acidity. Fermentation 5, 27. doi: 10.3390/fermentation5010027

[B70] Vilela A. Cosme F. Inês A. (2020). Wine and non-dairy fermented beverages: A novel source of pro-and prebiotics. Fermentation 6, 113. doi: 10.3390/fermentation6040113

[B71] Virdis C. Sumby K. Bartowsky E. Jiranek V. (2021). Lactic acid bacteria in wine: Technological advances and evaluation of their functional role. Front. Microbiol. 11. doi: 10.3389/fmicb.2020.612118 PMC7843464 33519768

[B72] Wang J. Ma Y. Sam F. E. Gao P. Liang L. Peng S.. (2022a). The impact of indigenous non-*saccharomyces* yeasts inoculated fermentations on ‘Semillon’Icewine. Fermentation 8, 413. doi: 10.3390/fermentation8080413

[B73] Wang S. Zhang Q. Zhao P. Ma Z. Zhang J. Ma W.. (2022b). Investigating the effect of three phenolic fractions on the volatility of floral, fruity, and aged aromas by HS-SPME-GC-MS and NMR in model wine. Food Chemistry: X 13, 100281. doi: 10.1016/j.fochx.2022.100281 35498990 PMC9040039

[B74] Yaa’ri R. Schneiderman E. Ben Aharon V. Stanevsky M. Drori E. (2024). Development of a novel approach for controlling and predicting residual sugars in wines. Fermentation 10, 125. doi: 10.3390/fermentation10030125

[B75] Yao M. (2023). Microbial diversity on grape surface and its research status. J. Eng. Sci. 2), 158–172. doi: 10.52326/jes.utm.2023.30(2).14

[B76] Zhang Y. L. Chen J. B. Lei Y. Zhou Q. Sun S. Q. Noda I. (2010). Discrimination of different red wine by Fourier-transform infrared and two-dimensional infrared correlation spectroscopy. J. Mol. Structure 974, 144–150. doi: 10.1016/j.molstruc.2010.03.021

[B77] Zhang Y. Kennedy J. F. Knill C. J. Panesar P. S. (2006). Kinetic analysis of beer primary fermentation using yeast cells immobilized by ceramic support adsorption and alginate gel entrapment. Artif. cells Blood substitutes Biotechnol. 34, 395–405. doi: 10.1080/10731190600769644 16818413

[B78] Zhu X. Torija M. J. Mas A. Beltran G. Navarro Y. (2021). Effect of a multistarter yeast inoculum on ethanol reduction and population dynamics in wine fermentation. Foods 10, 623. doi: 10.3390/foods10030623 33804257 PMC7998366

