# Supplementary material for: Influence of indigenous non-Saccharomyces yeast strains on the physicochemical and sensory properties of wine fermentation: a promising approach to enhancing wine quality
Source: Front Cell Infect Microbiol. 2024 Dec 6;14:1495177. doi: 10.3389/fcimb.2024.1495177 (PMC11659219; doi:10.3389/fcimb.2024.1495177)
Supplement: Supplementary file 1 [file Table1.docx]

**Supplementary Table 1: Initial screening of best wine fermenting strains**

| **Yeast strains** | **Fermenting ability (Phenol red broth test)** | | **Presence of buddings** | **Alcohol production ability (%v/v)** | **pH level** | **Biomass (CFU mL⁻¹.)** |
| --- | --- | --- | --- | --- | --- | --- |
|  | Color change | CO_2_ formation |  |  |  |  |
| J1Y | ++ | ++ | + | 7.57±0.01^p^ | 2.83±0.02^abc^ | 10^2^ |
| JSF3 | + | ++ | + | 6.15±0.01^de^ | 2.94±0.17^abcde^ | 10^1^ |
| Y5P | +++ | ++ | + | 7.33±0.06^o^ | 2.91±0.01^abcde^ | 10^1^ |
| JF3 | ++ | + | ++ | 8.15±0.01^s^ | 3.61±0.02^i^ | 10³ |
| JF11 | + | + | ++ | 6.94±0.03^n^ | 3.05±0.06^bcdef^ | 10^2^ |
| Y1P | ++ | + | + | 6.83±0.02^m^ | 3.29±0.03^fgh^ | 10^2^ |
| WMP4 | +++ | ++ | ++ | 7.53±0.03^p^ | 3.55±0.03^hi^ | 10³ |
| JU1 | + | + | + | 8.02±0.02^r^ | 3.22±0.03^efg^ | 10^2^ |
| JIP | + | + | ++ | 6.53±0.04^kl^ | 3.07±0.03^cdef^ | 10^1^ |
| UP12 | + | + | + | 6.03±0.03^c^ | 3.18±0.02^defg^ | 10^2^ |
| UP18 | + | + | + | 6.18±0.02^defg^ | 3.17±0.04^defg^ | 10^2^ |
| P12 | ++ | + | + | 6.13±0.03^d^ | 2.77±0.12^abc^ | 10^1^ |
| JF9 | + | + | + | 6.23±0.03^efg^ | 3.02±0.03^abcdef^ | 10^1^ |
| UP7 | + | + | + | 6.15±0.06^de^ | 2.94±0.03^abcde^ | 10^1^ |
| P4 | + | + | + | 6.48±0.04^jk^ | 2.89±0.04^abcde^ | 10^2^ |
| WM1 | + | + | + | 6.57±0.03^l^ | 2.72±0.03^ab^ | 10^2^ |
| J2P | + | + | + | 6.24±0.03^fg^ | 2.86±0.04^abcd^ | 10^2^ |
| UP3 | + | + | + | 6.80±0.03^m^ | 3.02±0.04^abcdef^ | 10^1^ |
| JF6 | + | + | + | 6.42±0.03^ij^ | 2.74±0.05^abc^ | 10^1^ |
| JFW | ++ | + | + | 6.34±0.02^hi^ | 2.88±0.04^abc^d | 10^1^ |
| PN2 | + | + | + | 6.27±0.03^gh^ | 3.08±0.07^cdef^ | 10^2^ |
| PN4 | + | + | + | 6.37±0.04^i^ | 3.03±0.04^abcdef^ | 10^1^ |
| JP5 | + | + | + | 5.24±0.03^a^ | 2.95±0.03^abcde^ | 10^2^ |
| WP1 | + | + | + | 5.93±0.05^b^ | 3.08±0.04^cdef^ | 10^2^ |
| Y8P | ++ | + | + | 7.67±0.04^q^ | 3.42±0.02^ghi^ | 10^2^ |
| J3P | + | + | + | 6.17±0.03^def^ | 2.84±0.03^abcd^ | 10^2^ |
| UP13 | + | + | + | 6.38±0.07^i^ | 2.92±0.03^abcde^ | 10^2^ |
| JPN | + | + | + | 6.26±0.03^fgh^ | 2.69±0.05^a^ | 10^1^ |

Data presented as mean ± standard deviation (n=3). Different alphabetic letters indicate significant differences between strains.
